# Research on the Interaction Mechanism and Structural Changes in Human Serum Albumin with Hispidin Using Spectroscopy and Molecular Docking

**DOI:** 10.3390/molecules29030655

**Published:** 2024-01-30

**Authors:** Si-Hua Fan, Wen-Qiang Wang, Yu-Wen Zhou, Xue-Jun Gao, Qiang Zhang, Ming-Hui Zhang

**Affiliations:** 1 College of Biology and Food Engineering, Guangdong University of Petrochemical Technology, No. 1, Kechuang Road, Maonan District, Maoming 525000, China; sihuafan@163.com (S.-H.F.); wwq980218@163.com (W.-Q.W.); 2 College of Animal Science and Technology, Yangtze University, 88 Jingmi Road, Jingzhou District, Jingzhou 434025, China; w21250511@163.com (Y.-W.Z.); gaoxj53901@163.com (X.-J.G.)

**Keywords:** human serum albumin, hispidin, fluorescence survey, molecular docking, spectrophotometry

## Abstract

The interaction between human serum albumin (HSA) and hispidin, a polyketide abundantly present in both edible and therapeutic mushrooms, was explored through multispectral methods, hydrophobic probe assays, location competition trials, and molecular docking simulations. The results of fluorescence quenching analysis showed that hispidin quenched the fluorescence of HSA by binding to it via a static mechanism. The binding of hispidin and HSA was validated further by synchronous fluorescence, three-dimensional fluorescence, and UV/vis spectroscopy analysis. The apparent binding constant (Ka) at different temperatures, the binding site number (n), the quenching constants (Ksv), the dimolecular quenching rate constants (Kq), and the thermodynamic parameters (∆G, ∆H, and ∆S) were calculated. Among these parameters, ∆H and ∆S were determined to be 98.75 kJ/mol and 426.29 J/(mol·K), respectively, both exhibiting positive values. This observation suggested a predominant contribution of hydrophobic forces in the interaction between hispidin and HSA. By employing detergents (SDS and urea) and hydrophobic probes (ANS), it became feasible to quantify alterations in Ka and surface hydrophobicity, respectively. These measurements confirmed the pivotal role of hydrophobic forces in steering the interaction between hispidin and HSA. Site competition experiments showed that there was an interaction between hispidin and HSA molecules at site I, which situates the IIA domains of HSA, which was further confirmed by the molecular docking simulation.

## 1. Introduction

Hispidin was first extracted from the sarcocarp body of the *Inonotus hispidus* fungus in 1889, and its structure was subsequently elucidated to be 6-(3,4-dihydroxyphenyl)-4-hydroxy-2-pyrone in 1961 [1,2]. As a secondary metabolite, hispidin is found in numerous fungus families, including *Hymenochaetaceae* [3], *Hymenogastraceae* [4], *Phaeolaceae* [5], *Omphalotaceae* [6], and *Cortinariaceae* [7]. Additionally, several plants, such as *Equisetum arvense* [8], *Pistacia atlantica* [9], *Leishmania amazonensis* [10], and *Goniothalamus umbrosus* [11], also contain hispidin and its derivatives [12]. Hispidin is attracting the attention of researchers for its potential in preventing and treating cancer [13], cardiovascular disease [14], neurodegenerative illnesses [15], and viral infections [16]. Hispidin has been shown to have anti-inflammatory, anti-oxidant, antiallergic, antiangiogenic, and hypoglycemic properties in earlier studies. Recently, the antidiabetic properties of hispidin have been observed to inhibit ferroptosis, thereby safeguarding pancreatic beta cells (PBCs) from damage induced by excessive glucose [17]. Although there are many potential health benefits associated with hispidin, further research is needed to determine whether or not it is safe for human usage.

The extent to which exogenous substances bind to blood albumin dictates their distribution and metabolism [18,19,20]. Through their reversible interaction with plasma proteins, such as human serum albumin (HSA), certain drugs, food additives, and other bioactive small molecules can alter their distribution, free concentration, and metabolism [21,22,23]. In addition, reducing aggregate formation and generally lengthening a drug’s half-life, HSA binding has exceptional effects on drug transport, release, and solubility [24]. HSA is one kind of soluble protein that is widely available in plasma; it contributes to 80% of osmotic blood pressure [25], and binds and moves naturally occurring hydrophobic ligands. HSA is a protein with 585 amino acid residues and a molecular weight of 66,500 Da [26]. It makes up around 4.5% of the mass of blood in humans and aids in regulating the pH and osmotic pressure of blood [27]. In a physiologically healthy state, its blood concentration falls between 0.5 and 0.75 mM (35 and 50 mg/mL) [28]. Three homologous domains, I (residues 1–195), II (residues 196–383), and III (residues 384–584), are present in HSA. Each domain is divided into two subdomains, A and B, which share sequence amino acids. Sudlow’s sites I and II are the main places where HSA combines, which reside in particular gaps in subdomains IIA and IIIA, respectively, and a few subordination places (site III or digitoxin sites), and are largely responsible for HSA’s ability to combine some aromatic and heterocyclic ligands [29]. Site I is a pocket that arises within subdomain IIA. It is also known as the warfarin-azapropazone site [30]. The lone tryptophan (Trp214) in the protein is found there. Subdomain IIIA contains site II, which has an affinity for ibuprofen. Hydrophobic amino acid residues are present in the interior pocket, whereas two crucial residues of amino acids (Arg410 and Tyr411) are present in the exterior pocket. Moreover, hemin is a site probe for domain I [31]. Additionally, other research has discovered that fatty acids can be used as probes to locate the binding site in HSA subdomain IIA [32].

In this work, the interaction between hispidin and HSA was investigated using various methodologies, including fluorescence quenching, synchronous fluorescence, three-dimensional fluorescence, UV/vis spectroscopy, hydrophobic probe assays, and site competition experiments. The quenching mode, the apparent binding constant, the quantity of binding sites, and the thermodynamic constants of the mutual effect of hispidin with HSA were determined. Finally, to validate the binding site and binding modalities of hispidin with HSA, a molecular docking simulation was performed. This work provides fundamental data to elucidate the binding mechanism of hispidin with HSA, which can aid in comprehending the pharmacological or toxicological effects of hispidin.

## 2. Results

### 2.1. Fluorescence Quenching Analysis

**Figure 1:**
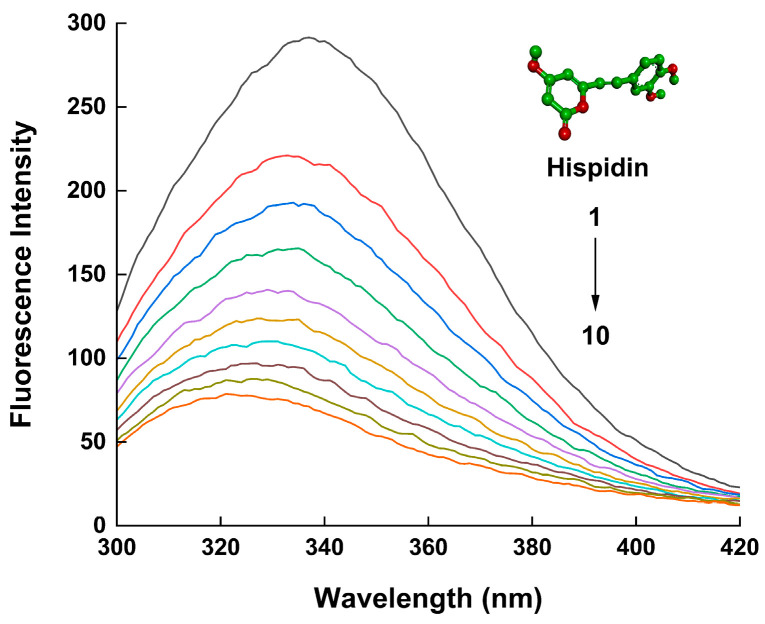
Fluorescence emission spectra (300–420 nm) of 5 µmol/L HSA in Tris–HCl buffer at pH 7.4, and 298 K, before and after the addition of hispidin, with excitation at 280 nm. The hispidin concentrations were 0, 5, 10, 15, 20. 25, 30, 35, 40, and 45 µmol/L from (1) to (10). The structure of hispidin is matched by the inset.

**Figure 2:**
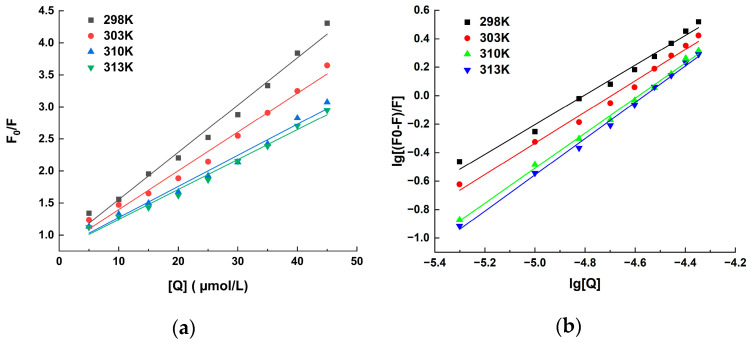
Stern–Volmer plots (**a**) and lg (F0 − F)/F plotted lg[Q] (**b**) illustrate HSA at temperatures of 298 K, 303 K, 310 K, and 313 K with varying hispidin concentrations.

Upon the occurrence of protein conformational changes, subunit interactions, substrate binding, or denaturation, there are frequent variations observed in the fluorescence emission spectra of fluorophores. Figure 1 displays the fluorescence spectra of the HSA solution with different concentrations of hispidin. The value of the HSA’s fluorescence intensity gradually decreased with the addition of hispidin, indicating that there is an interaction between hispidin and HSA. Illustrated in Figure 2a is the linear Sterne–Volmer plots of F0/F against Q. According to the relevant literature, if Kq is greater than 2 × 10^10^ L/(mol·s), the interaction between molecules is a static binding process [33]. According to Table 1, at various temperatures, the Kq values were more than 2 × 10^10^ L/(mol·s), illustrating the presence of a static quenching mechanism in the HSA–hispidin interaction. Static quenching arises from the formation of a non-fluorescent substance due to the interaction between the quencher and the fluorophore. Therefore, as the temperature increases, the system’s disorder intensifies, leading to a reduction in the Ksv value. This form of quenching does not primarily result from molecular collisions between hispidin and HSA; instead, it involves the formation of a complex when the fluorophore is excited.

According to the slope and intercept of the linear regression curve of lg[(F0 − F)/F] against lg[Q] in Figure 2b, the number of binding sites (n) and Ka in Table 2 can be obtained. All examined interactions exhibit n values very close to 1, indicating that hispidin can bind exclusively to a single binding site in HSA. The Ka is approximately equal to 10^5^ L/mol, suggesting that hispidin and HSA have a significant interaction. However, the results presented in Table 3 reveal that the Ka values for the hispidin–HSA interaction at 298 K decreased to 2.84 × 10^4^ and 3.09 × 10^4^ L/mol when SDS or urea was introduced. This indicates that SDS or urea has the potential to disrupt the hispidin–HSA interaction.

Table 2 further displays the calculated results for additional apparent thermodynamic parameters, encompassing entropy, enthalpy, and Gibbs free energy changes (∆H, ∆S, and ∆G). At the four temperatures, the ∆G values for the hispidin–HSA interaction are −28.60, −30.00, −33.47, and −34.80 kJ/mol, respectively, suggesting that the interaction between hispidin and HSA is thermodynamically favorable. In this study, both ∆H and ∆S were found to be greater than 0, indicating that hydrophobic forces are primarily responsible for hispidin’s binding to HSA.

### 2.2. Synchronous Fluorescence Analysis

**Figure 3:**
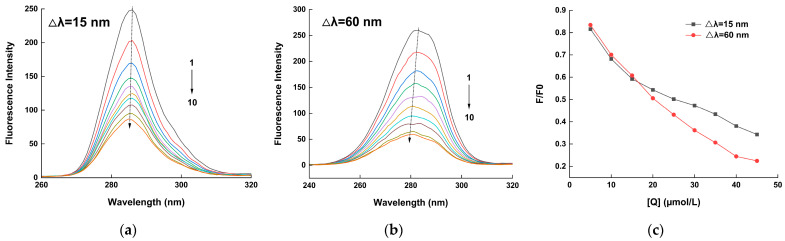
Synchronous fluorescence spectra. Δλ = 15 nm (**a**) and 60 nm (**b**) with hispidin concentrations of 0, 5, 10, 15, 20, 25, 30, 35, 40, and 45 μmol/L, respectively. Rate of fluorescence variation (**c**).

Insights into protein structural changes and the chromophore microenvironment can be derived from the synchronous fluorescence spectra. The tyrosine (Tyr) and tryptophane (Trp) residue polarity is revealed by the synchronous fluorescence spectra at wavelength differences (Δλ) of 15 nm and 60 nm, respectively [34]. As shown in Figure 3a, at Δλ = 15 nm, the fluorescence intensity of HSA decreased as the concentration of hispidin increased. These findings show that the microenvironment of Tyr residues was altered when hispidin and HSA were combined. Using Δλ = 60 nm in Figure 3b, it is evident that an observable blue shift in fluorescence curves and a notable decrease in HSA fluorescence intensity occurred with an increase in the concentration of hispidin. This suggests that the microenvironment of Trp residues in HSA was becoming more polar. Since the polarity of the surrounding environment affects the position shift of amino acid residues’ maximum emission wavelength, this information is frequently utilized to assess conformational changes in protein structure. In Figure 3c, it is also evident that the fluorescence value of Δλ = 60 nm declined more rapidly than that of Δλ = 15 nm.

### 2.3. UV/vis Spectroscopy Analysis

**Figure 4:**
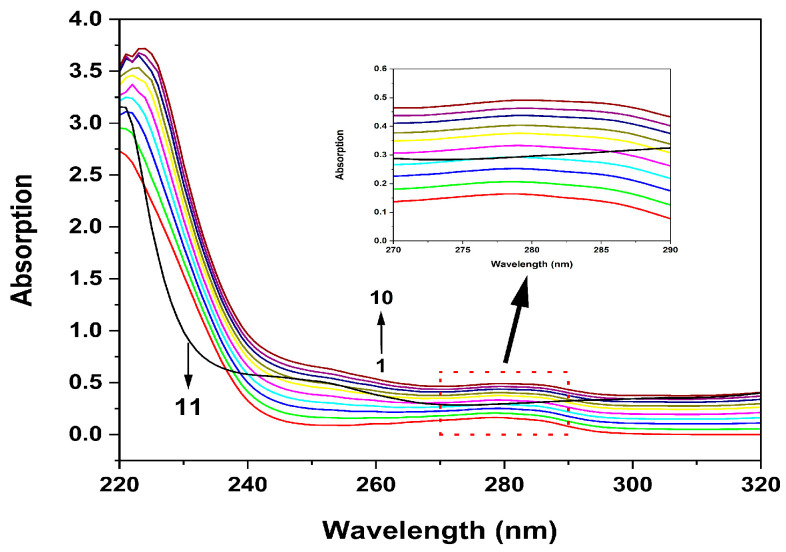
UV/vis absorption spectra of HSA. The level of HSA was 5 μmol/L and hispidin concentration was 0–45 μmol/L (1)→(10). Curve 11 is the UV spectrum of only hispidin with a concentration of 45 μmol/L.

Measuring UV/vis absorption is a very straightforward technique that can be used to investigate protein structural changes. A shift in the absorption can result from modifications to the microenvironment surrounding the chromophore molecules (Tyr, Trp, and Phe) in proteins [35]. π-π* electron transfer on the benzene heterocycle of tryptophan and tyrosine was identified due to the distinctive protein absorption peak at 280 nm [36]. Figure 4 displays the UV/vis absorption spectra of HSA in Tris-Cl buffer from 220 to 460 nm. With the gradual increase in hispidin concentration, HSA’s absorption of UV and visible light is greatly increased. However, a single solution of hispidin with a concentration of 45 μmol/L has no absorption peak at 280 nm. This study showed that the system produced the hispidin–HSA complex and that the hydrophobicity and polarity of the microenvironment surrounding the Tyr and Trp residues were impacted by the presence of hispidin.

### 2.4. Three-Dimensional Fluorescence Spectroscopy Analysis

**Figure 5:**
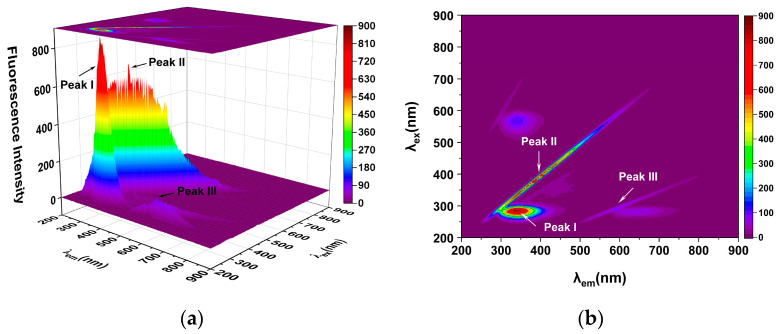

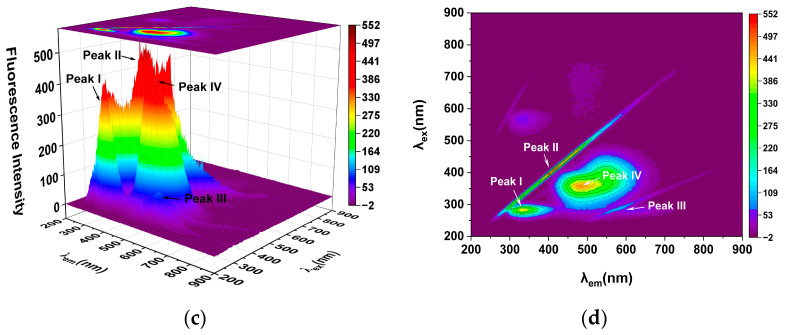
Three-dimensional fluorescence contour map of HSA (**a**,**b**) and the hispidin–HAS mixture (**c**,**d**). (**a**,**b**) 5 μmol/L HSA, 0 μmol/L hispidin; (**c**,**d**) 5 μmol/L HSA, 25 μmol/L hispidin; pH 7.4, at 298 K.

Additionally, three-dimensional fluorescence spectroscopy was used to look at the conformational behavior of HSA as it binds to hispidin. Figure 5 displays three-dimensional fluorescence maps and contour maps of the HSA solution and the hispidin–HSA mixture. Table 4 presents the matching characteristic peak values. The Rayleigh scattering peak was identified as peak II (ex = em) in Figure 5, while the second-order scattering peak was identified as peak III (em = 2 ex). These results show that peak I of the protein (HSA) moved from 340 nm to 332 nm with hispidin present, and the fluorescence intensity decreased significantly from 780.6 to 397.9. This indicates that the interaction between HSA and hispidin results in the structural alteration of HSA.

### 2.5. Hydrophobic Probe Assay and Hydrophobicity

**Figure 6:**
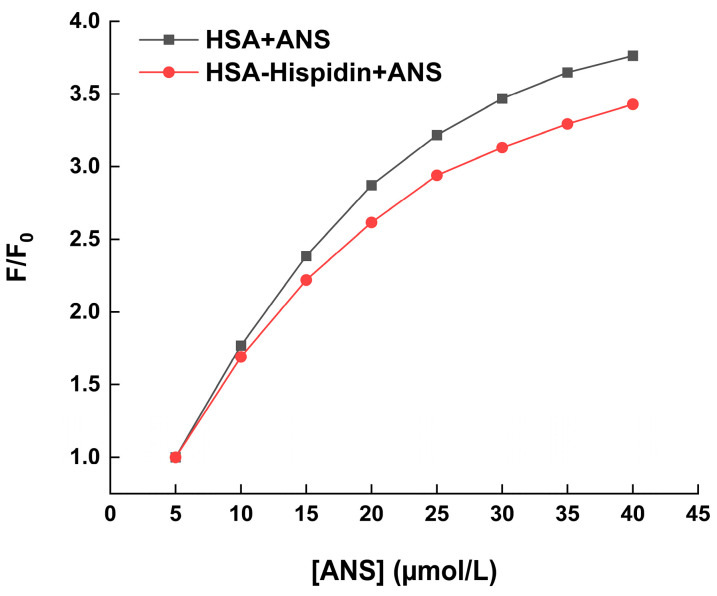
Effect of hispidin on the relative fluorescence of ANS in HSA solution.

The fluorescence dye ANS is sensitive to microenvironmental changes in proteins. Free ANS exhibits slight or no fluorescence, but a noticeable fluorescence signal will be produced as it attaches itself to the hydrophobic portion of proteins [37]. Figure 6 displays graphs of the relative fluorescence intensity (F/F0) against ligand concentration ([ANS]). The findings show that as ANS was added to the HSA with or without hispidin solution, the ANS relative fluorescence intensity rose. The results reveal that the relative fluorescence intensity increased with the addition of ANS to the HSA with or without hispidin solution, but the increase in relative fluorescence intensity was inhibited when hispidin was present in the HSA and ANS mixed system. This led to the prediction that the binding of hispidin would decrease the hydrophobic area of HSA, or that hispidin and ANS would competitively bind to the same hydrophobic region of HSA. The thermodynamic studies showed that hydrophobic force was crucial for maintaining the stability of hispidin–HSA complexes. As displayed in Table 5, an increase in hispidin concentration was accompanied by a progressive decrease in the *S*_0_ values of HSA, suggesting that the introduction of hispidin increased the occupancy of the hydrophobic cavity of HSA, consequently diminishing its hydrophobicity.

### 2.6. The Site Competition Analysis

To determine the binding site of hispidin on HSA, site marker competitive studies were conducted by utilizing medicines selectively attached to a recognized site or location on HSA. As mentioned above, warfarin, ibuprofen, digitoxin, and hemin exhibit a preference for binding with site I, site II, site III, and domain I in HSA, respectively. According to Equation (2), the Ka was assessed while the site markers were present, and Table 6 presents the findings. The results show that the Ka values of hispidin binding to HSA were decreased when ibuprofen or warfarin was present, and the latter decreased more, indicating that hispidin is more likely to bind to HSA at site I, which is situated in the hydrophobic cavity of IIA. However, the ibuprofen site cannot be excluded, it is possible that hispidin combines warfarin and ibuprofen at the location of the drugs. At the binding sites for ibuprofen (FA3, 4) and warfarin (FA7), hispidin is thought to bind competitively. Interestingly, hemin raises the Ka value, suggesting that hemin-bound HSA undergoes conformational changes that favor bound hispidin.

### 2.7. Molecular Docking Simulation

**Figure 7:**
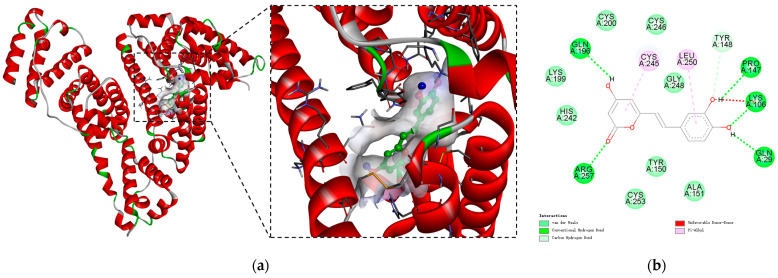
Molecular modeling of hispidin-bound HSA. (**a**) The optimal position for hispidin and HSA using the lowest amount of energy. (**b**) Particular location of hispidin and HSA combo in the 2D structural diagram.

According to the results of site competition experiments, hispidin preferred to connect to site I of HSA. Through molecular modeling simulations, the binding of hispidin at the HSA active site was discerned, providing an intuitive understanding of the interaction between the two. Following several pretreatments, the Discovery Studio program yielded the primary conformation of the hispidin–HSA complex. The simulated result of the predominant configuration of the hispidin–HSA complex is shown in Figure 7a, where the binding energy is the lowest, at −31.59 kJ/mol. It was discovered that hispidin mostly binds to site I or the IIA subdomain of HSA. Sixteen amino acid residues encircle the binding site, Cys200(A), Gln196(A), Lys199(A), His242(A), Arg257(A), Cys253(A), Ala151(A), Gln29(A), Lys106(A), Pro147(A), Tyr148(A), Cys246(A), Cys245(A), Leu250(A), Gly248(A), and Tyr150(A), as shown in Figure 7b. Hydrophobic interactions are the primary mechanism by which hispidin and the active amino acids Cys245(A) and Leu250(A) remain stable. In addition, the hydrogen atoms on the amino acid residues Gln196(A), Arg257(A), Gln29(A), Lys106(A), and Pro147(A) form bonds with the hydroxyl oxygen atoms on the benzene ring of hispidin. According to the reference, the amino acids that determined the coordinates of site-specific docking were as follows: for site 3 (sub-domains IIIA and IIB), Ser-342, Arg-348, and Arg-485; for site 4 (subdomain IIIA), Arg-410, Tyr-411, Ser-489, Ser-419, and Thr-422; for site 6 (sub-domains IIA and IIB), Arg-209, Lys-351, and Ser-480; and finally, site 7 (subdomain IIA) included Lys-199, Arg-218, Arg-222, His-242, and Arg-257 [38,39,40]. Based on our docking results, hispidin binds to FA7 (warfarin binding site), where we located interactions with Lys-199, His-242, and Arg-257. Unfortunately, no FA3 and FA4 sites were found. In this study, the results of site competition and thermodynamic analysis are supported by molecular docking, providing mutual corroboration.

## 3. Discussion

Vitamins, hormones, steroids, fatty acids, and other endogenous and exogenous compounds (drugs, toxins, phytochemicals) are among the diverse substances that HSA is essential in binding and conveying. Certain small compounds included in food have the ability to bind to HSA and change its conformation or spatial configuration. On the contrary, HSA may have an influence on small molecule metabolism and their effective concentration in the body. Consequently, research on the interaction between hispidin and HSA is crucial for the fields of chemistry, medicine, biology, and food nutrition and health.

Due to its great sensitivity, simplicity in use, and speed, fluorescence spectroscopy is a commonly used method for determining non-covalent interactions between proteins and small molecules [41]. The static and dynamic quenching mechanisms are distinguished according to temperature dependence [42]. In this study, hispidin exhibited the ability to quench the fluorescence of HSA, and the static quenching characteristic was quantified using the Ksv value, which consistently decreased with an increase in temperature. This effect aligns with previous observations during the interaction between 4-(1h-indor-3-yl)-2-(ptolyl) quinazoline-3-oxide and human serum albumin [43]. Another method to investigate the quenching process is to utilize the maximal biomolecular scattering collision quenching constant (Kq). A drop in fluorescence intensity induced by static quenching occurs when the Kq value exceeds 2 × 10^10^ L/(mol∙s). Otherwise, the dynamic quenching of protein binding occurs. The information in Table 1 demonstrates that the Kqs ranged from 7.37 to 4.67 × 10^12^ L/(mol·s) at various temperatures, all of which were higher than 2 × 10^10^ L/(mol·s), also suggesting that the static quenching process is responsible for the interaction between hispidin and HSA. Tryptophan (Trp) and tyrosine (Tyr) residues are the primary sources of intrinsic fluorescence in proteins. These residues can be quenched following interactions with ligands, which alters the protein’s intrinsic fluorescence intensity [44]. The characteristic information of Tyr or Trp is determined through synchronous fluorescence when the wavelength intervals (Δλ) are stabilized at 15 nm or 60 nm, respectively [45]. In this study, the synchronous fluorescence spectra showed that adding hispidin caused a decrease in the fluorescence intensity of HSA (Figure 3). The results suggest that the microenvironments of Tyr and Trp undergo changes due to chemical interactions, leading to the hispidin-induced fluorescence quenching of HSA.

Hispidin is a phenolic chemical that is a member of the C=C-bond-type bicyclic aromatic compounds [46]. Under non-oxidizing conditions, phenolic compounds and plasma proteins form reversible complexes are mediated by hydrogen bonding, electrostatic interactions, hydrophobic effects, and van der Waals forces [47]. Thermodynamic characteristics like ∆H and ∆S influence the way phenolic compounds interact with proteins. Hydrophobic interaction is indicated by positive values of both parameters; van der Waals forces and hydrogen bonds are indicated by negative values; and electrostatic forces are significant in aqueous solutions, as indicated by ∆S > 0 and ∆H < 0. In the results of this study, both ∆S and ∆H are positive, suggesting that the hydrophobic interaction between the aromatic ring of hispidin and the hydrophobic amino acid residues of HSA is the primary force stabilizing the hispidin–HSA interaction. In the interaction between the albumins and chemicals, the aromatic ligands are important. Hydrophobic linkages are created when the aromatic ring of a polyphenol is attracted to hydrophobic areas of other substances. If the complexes have a more hydrophobic character due to the presence of aromatic ligands, the hydrophobic interaction between the complexes and albumins is prominent. In hispidin with two aromatic rings, it makes sense to think of hydrophobic interactions as the main types of interactions. The two main non-covalent forces involved in phenol–protein interactions are often hydrogen bonding and hydrophobic interactions. This is consistent with previous findings that the binding between naringenin or genistein and HSA is strongly involved in hydrophobic interactions [48,49]. To verify the hydrophobic interaction between hispidin and HSA, we also employed SDS and urea as the reagents. As urea functions as a hydrogen bond receptor, it can break both hydrophobic and hydrogen bonds in proteins, causing the proteins to unfold, whereas hydrophobic connections can be entirely or partially broken by SDS, an anionic detergent [50]. Therefore, the hydrogen bonds and hydrophobic interactions between ligands and proteins can be broken by urea and SDS, respectively. The information in Table 3 demonstrates that urea and SDS reduced the Ka of hispidin–HSA, indicating that hydrophobic interactions are crucial for hispidin’s binding to HSA.

Due to its high affinity for the hydrophobic surface of proteins, ANS is used as a probe for hydrophobic fluorescence. It is also used as a microenvironment probe for proteins because of its exceptional capacity to exhibit peak shifts and intensity variations in response to the solvent environment in which it is found [51]. A more thorough explanation of the hydrophobic clusters can be obtained from the fluorescence of ANS attached to proteins [52]. In this study, the role of hydrophobic force in the hispidin–HSA interaction was proven again by employing ANS to measure the surface hydrophobicity (*S*_0_) of HSA (Table 5).

To further explore the binding position of the hispidin–HSA interaction, we conducted a site-competition test and molecular docking simulation. It can be seen from Table 6 that when ibuprofen and warfarin are present, the Ka value is reduced by 32.5% and 42.7%, respectively (Table 6). Large-volume heterocyclic anions with charges located at the center of the molecule (e.g., warfarin) typically attach to site I of the HSA, while site II is where aromatic carboxylic acids, like ibuprofen, that have prolonged conformations and negative charges at one end of the molecule interact, which can be found in the hydrophobic cavities of HSA subdomains IIA and IIIA, respectively [53]. Thus, hispidin binds to ibuprofen and warfarin in the same location, particularly occupying site I of the warfarin-bound IIA subdomain. In domain IIA, the outcomes of molecular docking were also displayed, and the primary amino acid residues Cys245(A) and Leu250(A) were crucial to the interaction process between HSA and hispidin (Figure 7). This also confirms that the binding between hispidin and HSA is hydrophobic. Hispidin belongs to the styrene-pyranone family of compounds, which have similar effects to flavonoids in plants [54]. For instance, flavonol primarily binds to the IIA subdomain in HSA, and quercetin binds to a sizable hydrophobic cavity within the IIA subdomain, as indicated by a computational map of potential binding locations [55]. Curcumin and genistein primarily bind within the hydrophobic pocket at site I of Tyrosine 214 [56]. Gallic acid was found to be able to bind to HSA’s site I in a different investigation [57]. Phenolic compounds typically undergo high first-pass metabolism. Further investigation is warranted in the future to explore the interaction between hispidin primary metabolites and HSA.

## 4. Materials and Methods

### 4.1. Materials

HSA was purchased from Solarbio Biotechnology Co. Ltd. (Beijing, China). Hispidin (purity ≥ 98%), digitoxin (purity ≥ 98%), hemin (purity 98%), ibuprofen (purity ≥ 98%), warfarin (purity ≥ 98%), and 8-anilino-1-naphthalenesulfonic acid (ANS, purity 96%) were obtained from Macklin Biochemical Technology Co. Ltd. (Shanghai, China). Digitoxin, hemin, ibuprofen, and warfarin were used for binding site exploration. SDS and urea were purchased from Biosharp Reagent Co. Ltd. (Hefei, China) and Aladdin Biochemical Technology Co. Ltd. (Shanghai, China), respectively. The remaining reagents used were of analytical grade.

### 4.2. Preparation of Reaction Solutions

The concentration of Tris-HCl buffer solution was 0.2 mol/L, which included 0.1 mol/L NaCl, and the value of pH was kept at 7.4. Prior to use, the HSA stock (0.2 mmol/L) was diluted with Tris-Cl buffer solution, and the final HSA concentration was 5 µmol/L. In order to generate 40 mmol/L solutions, hispidin was dispersed in DMSO and maintained in a brown bottle. The interaction between hispidin and HSA was investigated using various methodologies, including fluorescence quenching, synchronous fluorescence, three-dimensional fluorescence, UV/vis spectroscopy, hydrophobic probe assays, and site competition experiments. Before the experiment, the stock solution was diluted with Tris-HCl to generate a working solution (5 mmol/L). The working hispidin solutions were added to a solution of HSA to generate different hispidin concentrations (5–45 µmol/L), with the DMSO content at the end being less than 0.01%. Additionally, to confirm how SDS and urea affect the interaction between HSA and hispidin, SDS and urea, which had been dissolved in Tris-Cl, were added to the HSA–hispidin mixed solution at final concentrations of 4 mol/L and 5 mmol/L, respectively. The fluorescence intensity values of the samples were measured in quartz glass cuvettes after a 5 min reaction period at four different temperatures (298, 303, 310, and 313 K).

### 4.3. Fluorescence Spectroscopy Analysis

Using an F97XP fluorescence spectrophotometer (Shanghai, China), the fluorescence spectrum of the HSA solutions, either with or without hispidin, SDS, and urea, was examined. The HSA concentration in all the experiments was 5 µmol/L. The emission spectra were obtained in the wavelength range of 300–420 nm using an excitation wavelength of 280 nm and a scanning speed of 1000 nm/min. The fixed slit widths for both emission and excitation were set at 5 nm. Tris-Cl buffer solution was used as a blank before the measurement.

#### 4.3.1. Identification of the Fluorescent Quenching Process

Two mechanisms were considered for the process of quenching fluorescence, which is the reduction in a fluorophore’s intensity: static and dynamic quenching. The mechanisms underlying fluorescence quenching were elucidated by employing the Stern–Volmer Equation (1) [58].

F0/F = 1 + Ksv·[Q] = 1 + Kq·δ0·[Q](1)

In Equation (1), F0 and F represent fluorescence intensity values without and with a quencher, respectively, Ksv stands for the quenching constant of Stern–Volmer, and Kq denotes the rate constants of quenching. Without a quencher, δ0 represents a molecular fluorescence lifetime of 10^−8^ s [59], and [Q] is the quencher concentration. A plot of F0/F versus [Q] was used to obtain Ksv from the slope of linear regression. In cases when the Kq value exceeds 2 × 10^10^ L/(mol·s), static quenching is the reason for the decreased fluorescence intensity; if not, there is dynamic quenching for the binding of the quencher protein.

#### 4.3.2. Ka Measurement and Site Numbering

During static quenching, small molecules individually bind to specific sites on a macromolecule [60]. To obtain the binding sites and Ka value between hispidin and HSA, the Lineweaver–Burk equation was created by modifying the Stern-Volmer equation (Equation (2)) [61].

lg[(F0 − F)/F] = lgKa + n·lg[Q](2)

In this formula, the fluorescence intensity when the quenching agent is present is F, and when it is not, it is F0, and [Q] is the concentration of the quenching agent.

#### 4.3.3. Determining Evident Thermodynamic Parameters

The Van ’t Hoff equation (Equation (3)) was utilized to determine the apparent enthalpy and entropy changes (∆H and ∆S) by analyzing the slope and intercept of the lgKa versus 1/T curve; however, using Equation (4), the standard free energy change G^◦^ of hispidin binding to HSA was computed [62].

In Ka = −∆H/(RT) + ∆S/R(3)

∆G = −RT ln Ka(4)

R is the gas constant, which is 8.314 J/(mol K), and T stands for thermodynamic temperature, also known as the absolute temperature, T = t (°C) + 273.15. Ka is calculated from Formula (2). Generally, non-covalent interactions between small molecules and biological protein macromolecules involve four non-covalent interaction forces. The hydrophobic force acts as the primary driving force when ∆H > 0 and ∆S > 0; Van der Waals and hydrogen bonding forces predominate if ∆H < 0 and ∆S < 0; however, the contact is controlled by electrostatic force if ∆H < 0 and ∆S > 0 [63].

### 4.4. Synchronous Fluorescence Analysis

The alterations in luminous amino acids during the interaction of proteins with small molecules were observed using synchronous fluorescence spectroscopy. Based on the equation Δλ = λem − λex, the spectral properties of protein tyrosine and tryptophan residues are displayed in the fluorescence spectra at Δλ = 15 nm and Δλ = 60 nm, respectively. In this paper, the fluorescence spectra of HSA interacting with hispidin were scanned using this method. The fixed slit widths for both emission and excitation were set at 5 nm.

### 4.5. UV/vis Spectroscopy Analysis

Different hispidin concentrations (0–45 µmol/L) were obtained by combining the HSA solution (5 µmol/L) with the hispidin working solutions and carrying out a reaction at room temperature for 5 min. The UV absorption peak at 220–460 nm was measured for the HSA mixes and hispidin (45 µmol/L, without HSA) with a 0.1 nm sampling interval. Before the measurement, the baseline of the device was adjusted using the Tris-HCl buffer.

### 4.6. Three-Dimensional Fluorescence Analysis

With or without an equimolar hispidin concentration, the three-dimensional fluorescence spectra of the HSA were recorded at a scanning rate of 48,000 nm/min at 298 K. A range of 200–900 nm was employed for both the emission and excitation wavelengths. Ten nanometers was the fixed slit width for both the emission and excitation.

### 4.7. Hydrophobic Probe Assay and Hydrophobicity Measurements

In the hydrophobic probe analytical experiments, the first system maintained a constant HSA level of 5 µmol/L, while in the second system, the concentration ratio of the HSA–hispidin mixture was 1:3. Subsequently, increasing titration of the ANS solution (5 × 10^−3^ mol/L) was added to the first system or second system solutions. The ANS concentration varied from 5 to 45 µmol/L. In the hydrophobicity experiments, initially, three systems of compounded solutions were prepared with concentration ratios ([hispidin]/[HSA]) of 0, 1, and 3, respectively. Subsequently, a 1 μL mixed solution was titrated into the 1 mL ANS solution (9 × 10^−7^ mol/L) sequentially. Following each addition, all experiments were allowed to react for five minutes in the dark. The excitation wavelength for the ANS fluorescence spectra was 370 nm, and the measurements were conducted within the wavelength range of 400 to 600 nm. Lastly, a plot was generated using the HSA concentration against the fluorescence intensity value at 480 nm, where the slope of the initial segment signified the surface hydrophobicity (*S*_0_) of HSA.

### 4.8. Binding Site Exploration

To determine where hispidin binds to HSA, site competition experiments were performed on the interactions between proteins, probes, and hispidin. The fluorescence titration approach was used to carry out binding location experiments between hispidin and HSA with warfarin, ibuprofen, digitoxin, and hemin. The concentration ratio of HSA and site markers was 1:3, and a full reaction was performed at room temperature for 30 min; then, hispidin was added dropwise to the site marker–HSA mixtures. A 280 nm excitation wavelength was chosen and the fluorescence emission spectra of HSA were collected.

### 4.9. Molecular Docking Simulation

Using Autodock 4.2, Autodock tools (ADT) software and the Lamarckian genetic algorithm were used to fit the combination of hispidin and HSA. The stereochemical structure of hispidin (CID: 54685921) and the crystal structure of HSA (PDB id: 1H9Z) were retrieved from PubChem and the Brookhaven Protein Data Bank, respectively. During the docking process, the polar hydrogen bonds and Gasteiger charges of hispidin were inserted using the AutoDock tool. A deprotonated charged state was adopted for each ligand. In order to identify potential hispidin binding sites on proteins, grid boxes of 116 Å × 126 Å × 126 Å were chosen for blind docking the search areas that encompassed the entire HSA molecule. The parameters that were set were as follows: 150, 100, 2,500,000, and 2700 for the genetic algorithm (GA) population size, number of GA runs, maximum number of energy evaluations, and maximum number of generations. Following the completion of the docking computation, Discovery Studio 4.5 software was used to examine the optimal conformation, which had the lowest binding energy.

## 5. Conclusions

For the first time, we investigated in this study the relationship between hispidin and HSA using a variety of spectral techniques in conjunction with molecular docking simulations. From UV/vis and fluorescence spectra, it was discovered that the binding of hispidin to HSA induced diverse structural alterations in the protein. The results of the fluorescence quenching experiment demonstrated that the quenching ability of hispidin on HSA was static, and the fluorescence of HSA was quenched by the interaction of hispidin and HSA. Both SDS and urea can reduce the Ka by breaking hydrophobic interactions and hydrogen bonds. The results of the ANS substitution, site competition experiments, and molecular docking showed that hydrophobic interaction in the IIA domain was the combination site of hispidin on HSA. This work advances our understanding of the pharmacology and toxicity of hispidin as a food additive, as well as the potential applications of hispidin in dietary supplements or therapies.

## Figures and Tables

**Table 1:** The linear formulas, Ksv, and Kq for the interaction of hispidin with HSA at various temperatures.

T (k)	Equation	Ksv (10^4^ L/mol)	Kq (10^12^ L/(mol·s))	R^2^
298	Y = 0.0737Q + 0.8165	7.37	7.37	0.98
303	Y = 0.0605Q + 0.7929	6.05	6.05	0.98
310	Y = 0.0485Q + 0.7882	4.85	4.85	0.98
313	Y = 0.0467Q + 0.7773	4.67	4.67	0.98

**Table 2:** The apparent thermodynamic parameters and the apparent binding parameters (Ka) for the interaction between hispidin and HSA at various temperatures.

T (k)	Ka (10^5^ L/mol)	n	∆H (kJ/mol)	∆S (J/(mol·K))	∆G (kJ/mol)
298	1.03	1.04	98.75	426.29	−28.60
303	1.42	1.09			−30.00
310	4.36	1.22			−33.47
313	6.43	1.27			−34.80

**Table 3:** Impact of urea and SDS at 298 K on Ka of hispidin–HSA interaction.

Protein Complex	Ka (L/mol)
HSA + Hispidin	10.30 × 10^4^
HSA + Hispidin + SDS	2.84 × 10^4^
HSA + Hispidin + Urea	3.09 × 10^4^

**Table 4:** Three-dimensional fluorescence spectral characteristics of HSA and hispidin–HSA complexes.

System	Parameter	Peak II	Peak I
HSA	Peak position Δλex/Δλem (nm/nm)	350/350	280/340
	Intensity F	410.7	780.6
Hispidin–HSA	Peak position Δλex/Δλem (nm/nm)	350/350	280/332
	Intensity F	207.6	397.9

**Table 5:** The impact of hispidin on the surface hydrophobicity of HSA.

Molar Ratio [Hispidin]/[HSA]	*S*_0_	R^2^
0:1	15.72	0.96
1:1	14.87	0.97
3:1	10.03	0.98

**Table 6:** The effects of site markers on Ka of hispidin–HSA interaction.

System	Ka (10^5^ L/mol)	R^2^
HSA + hispidin	1.299	0.9932
HSA + hispidin + ibuprofen	0.877	0.9971
HSA + hispidin + warfarin	0.745	0.9901
HSA + hispidin + digitoxin	1.576	0.9960
HSA + hispidin + hemin	2.495	0.9965

## Data Availability

All data are contained within the article.
